# Medical and pharmacological approach to adjust the salbutamol anti-doping policy in athletes

**DOI:** 10.1186/s12931-015-0315-2

**Published:** 2015-12-24

**Authors:** Fabien Pillard, Michel Lavit, Valérie Lauwers Cances, Jacques Rami, Georges Houin, Alain Didier, Daniel Rivière

**Affiliations:** Respiratory Function Exploration and Sport Medicine Department, Larrey Hospital, 24 Chemin de Pouvourville, TSA 30030, 31059 Toulouse Cedex 9, France; Exercise Physiology Department, Medical School, Paul Sabatier University, Toulouse, France; INSERM, U858-Adipolab Unit, Institute of Molecular Medicine, Toulouse, France; Pharmacokinetic and Toxicologic Laboratory, Institute of Biology, Purpan Hospital, Toulouse, France; Epidemiology Department, Medical School, Paul Sabatier University, Toulouse, France; Department of Respiratory Diseases, Larrey Hospital, Toulouse, France

**Keywords:** Salbutamol, Sport, Doping, Beta2 agonist, Control

## Abstract

**Background:**

Salbutamol abuse detection by athletes is based on a urinary upper threshold defined by the World Anti-Doping Agency (WADA). However, this threshold was determined in healthy, untrained individuals and after a dose of salbutamol inhaled that might not really mirror the condition of asthmatic athletes and the experts’s guidelines for asthma management. We aimed to revise this threshold in accordance with recommended clinical practice (that appear to be different from the actual WADA recommendation) and in exercise conditions.

**Methods:**

For the present open-label design study, we included 12 trained male cyclists (20 to 40 y/o) with asthma. Differently from the previous pharmacokinetic study supporting the actual salbutamol urinary upper threshold, we decided to administer a close to recommended clinical practice daily dose of 3x200 μg.d^−1^ inhaled salbutamol (instead of 1600 μg.d^−1^ as authorized by the anti-doping policy). Urine salbutamol concentration was quantified by liquid chromatography-tandem ion trap mass spectrometry and corrected for urine density, at rest and after a 90-min cycling effort at 70-80 % of the maximal aerobic power.

**Results:**

The maximum urine salbutamol concentration value peaked after the cycling effort and was 510 ng.mL^−1^. That is twice lower than the actual WADA threshold to sanction salbutamol abuse, this “legal” threshold being based on pharmacokinetic data after a daily dose that is 8 fold the total dose sequentially administrated in our study. Considering its 95 % confidence interval, this threshold value could be more stringent.

**Conclusion:**

By using conditions in accordance with the experts’ clinical and safety guidelines for asthma management in athletes undergoing an intense exercise bout, our study suggests that the urine salbutamol concentration threshold could be lowered to redefine the rule supporting the decision to sanction an athlete for salbutamol abuse.

## Background

Although the efficacy of anti-doping controls has improved, fighting against doping remains a priority in sport policy. As exposed in the Final Revised 2015 World Anti-Doping Code edited by the World Anti-Doping Agency (WADA), prevention (ethic and health public aspects), detection (doping control) and sanction are key aspects of anti-doping policies [1].

The WADA list [1] of prohibited substances is accepted worldwide and has been adopted by the International Olympic Committee (IOC). For instance, beta2-adrenergic receptor agonist (BA) are listed as banned substances because they can be used for athletic performance enhancement [2–4]. However, BA are listed as one the medications to prevent and treat the symptoms of exercise-induced bronchospasm (EIB) among athletes, with or without a known diagnosis of asthma. Accordingly, athletes are sanctioned for BA use except when taking salbutamol, salmeterol and formoterol, for medical reasons, by inhalation, at a maximum dose and the urine concentration must not exceed a threshold value for salbutamol and formoterol.

Salbutamol is one of the most popular short-acting BA (SABA) used to relieve asthma symptoms. This medication can prevent asthma symptoms and should be taken 10 to 15 min before exercise. It will help prevent symptoms for up to four hours. Salbutamol can also be used to treat and reverse the symptoms of EIB. Based on the current WADA guidelines, the SABA salbutamol can be prescribed up to 1600 μg per 24 h. According to recent expert clinical guidelines from Global Initiative for Asthma (GINA) recommendations [5] and the American Thoracic Society (ATS) Clinical practice guidelines [6], SABAs can be prescribed during sport practice to prevent EIB, just before exercise: inhaled doses of 200–400 μg could be inhaled just before exercise as suggested by Tan et al. [7]. In addition, doses between 400 to 800 μg can be inhaled up to the 2nd or 4th hour of asthma attack, and then repeated if necessary but this situation should be considered with caution among athletes because acute and intense exercise triggers EIB [8]. Anyway, inhalation of 1600 μg is neither encouraged in a day for prevention of EIB. To justify the WADA daily upper limit of 1600 μg.d^−1^ for inhaled salbutamol (a limit that is 2 · 5 fold higher than the limit proposed by experts in asthma), Dickinson et al. suggested that this limit was proposed by WADA to take into account the fact “that asthmatic athletes are often instructed to use their inhaler on an *as needed basis* that could be interpreted by the athletes as a clearance to inhale unlimited amounts of salbutamol to combat respiratory symptoms” [9]. However, this assertion is in contradiction with experts guidelines cited above (GINA and ATS recommendations) and with recommendations published in a very recent review published by Boulet et al. [10]. According to these guidelines and recommendations, SABAs should be used only as-needed at the lowest dose and frequency required to prevent eventual tachyphylaxis. Moreover, this assertion could be discussed based on clinical practice and ethical considerations. Indeed asthma requiring such high BA doses should not be compatible with current high level physical and sports performance if we consider that the high levels of ventilation sustained in such exercise conditions are identified as triggers for EIB [2].

A second question concerns the maximum urine concentration of salbutamol accepted in the case of “therapeutic prescription”. Historically, salbutamol was the first bronchodilatator listed in the international anti-doping list according to a urinary threshold for sanction. According to Dickinson [9], this current WADA urinary threshold (1000 ng.mL^−1^) is based on data published in 2000 by Berges et al. [11]. In this study, non-asthmatic recreational swimmers inhaled 1600 μg salbutamol and then the urine salbutamol concentration was determined at rest. This study, like all the other studies consecutively published with the objective of assessing salbutamol pharmacokinetics after inhalation or oral administration (Table 1), was not conducted in asthmatic competitive athletes and samples were not collected during exercise. Therefore, it can be concluded that no study was conducted in laboratory conditions mirroring the exercise conditions experienced by asthmatic athletes. In addition, none of the studies on salbutamol was performed using an adjusted and clinical relevant dose of salbutamol to manage asthma or EIB in athletes (i.e., a dosing regimen in accordance with experts’ recommendations in the field).

**Table 1:** Published studies on the pharmacokinetics of inhaled salbutamol (updated on the 31st July 2014)

	Physical activity	Asthmatic status	Salbutamol inhaled dose	Rest or exercise	Exercise intensity	Sample size
Anderson 1998 [26]	Untrained	Healthy	1 × 180 μg	Rest		10
Berges 2000 [11]	**Trained**	**Asthmatic**	1 × 200 μg	Rest		**15**
		Healthy	4 × 400 μg			**17**
Pichon 2006 [27]	**Trained**	Healthy	**3 × 200 μg**	Rest		10
Sporer 2008 (1) [28]	**Trained**	Healthy	1 × 200 μg	**Exercise (and Rest)**	Cycling time	**30**
			and 1 × 400 μg			
			and 1 × 800 μg			
Sporer 2008 (2) [29]	Untrained	Healthy	1 × 200 μg	Rest		8
Elers 2010 [30]	Untrained	**Asthmatic**	1 × 200 μg	Rest		10
Elers 2011 [31]	Untrained	**Asthmatic**	4 × 400 μg	Rest		10
		Healthy				10
Elers 2012 [19]	**Trained**	**Asthmatic**	1 × 800 μg	Rest		10
		Healthy				
Dickinson 2014 [9]	**Trained**	Healthy	1 × 800 μg	**Exercise (and Rest)**	Not specified	**32**
			and 1 × 1600 μg			
**Theoretical “Link-field study”**	**Trained**	**Asthmatic**	**Preventive dose:** **3 × 200 μg**	**Exercise (and Rest)**	**Exercise**	**Powerful**

Bold data identify conditions that we consider as optimal based on a rigorous clinical and scientific approach

Accordingly, the objective of our study was to assess the pharmacokinetics of inhaled salbutamol 1) in asthmatic athletes and 2) after a single bout of intense exercise and 3) after a recommended (by experts in asthma but not according to the WADA list) dosing regimen of inhaled salbutamol for athletes undergoing an intense exercise bout (i.e. 200 μ.d^−1^ of salbutamol three times per day).

## Methods

### Study design

**Fig. 1:**
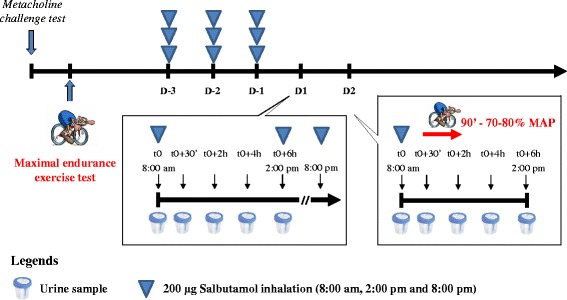
Study design

This study used an open-label design. All participants visited our laboratory four times. During the first visit the diagnosis of asthma was confirmed by direct bronchial provocation test with methacholine chloride. During the second visit, the endurance capacity was assessed to determine the intensity of the exercise session. Then, two consecutive sessions were dedicated to assessing salbutamol pharmacokinetics in plasma and urine at rest (rest pharmacokinetic session, D1) and, the next day, during a bout of intense endurance exercise (exercise pharmacokinetic session, D2). Participants were asked to stop their current anti-asthmatic treatment three days before the rest pharmacokinetic session and to inhale salbutamol three times per day (see the *Medications* section; study design is presented in Fig. 1).

The study protocol was approved by the scientific committee of the French Anti-Doping Agency and by the Toulouse hospital ethics committee (agreement n° 0830103).

### Participants and lung function assessment

This study included 12 men, aged between 20 and 40 year. They were trained cyclists and were enrolled in the study if they practiced eight hours or more of cycling per week. All were enrolled in competition. All were non smokers. All experienced clinical symptoms of asthma according to GINA classification, including EIB and all had been using a SABA on demand for at least one year. All the patients used SABA at least one time during the two weeks before their enrollment in the study. Patients reported they used SABA some days before exercise when they observed a “short breath” compared to other days. No one had ever had a severe asthma attack. In the absence of exacerbations, our cohort was defined as a mild asthma cohort.

Only men were studied in this preliminary study to exclude any pharmacological variation related to menstrual hormonal disturbances in women.

Written informed consent was obtained by all subjects.

The methacholine challenge test was used to confirm the diagnosis of asthma and a negative challenge test was used to exclude the diagnosis of asthma. All the participants were asked to stop their SABA at least 48 h before lung function assessment [12]. Pulmonary function testing was done using a whole body plethysmograph (Jaeger Master Screen Body™ Plethysmograph). The methacholine challenge test was performed using the aerosol provocation system (APS) dosimeter technique (APS, Viasys) [13]. The cumulative dose of methacholine provoking a 20 % decrease (PD20) in the forced expiratory volume in 1 s (FEV1) was calculated to confirm the participant’s asthmatic status. According to the ATS guidelines, the PD20 threshold was set at 1600 μg [12, 13].

### Endurance exercise testing and endurance exercise during the exercise pharmacokinetic session

The participant’s endurance capacity was assessed to check their fitness level. To this aim, maximal graded exercise tests were conducted in our laboratory. Subjects used their own bicycle and equipment. The power output was assessed using the Power Tap mobile cycling ergometer® (Cycle Ops, Madison, WI, USA) [14]. During the test, oxygen consumption (VO_2_, expressed in L.min^−1^ and mL.min^−1^.kg^−1^) was assessed using an Oxycon Pro ergospirometer® (Erich Jaeger, Viasys Healthcare, Germany). The VO_2_max and its corresponding power, the maximal aerobic power (MAP) were measured.

During the exercise pharmacokinetic session, subjects performed a 90 min bout of endurance exercise in the same general conditions as for the MAP determination (i.e., on their own bicycle and power output was assessed by using the Cycle OPS Power Tap™ system). After 10-min warm up at 60 % of their MAP, participants were asked to exercise at 70–80 % of their MAP for the last 80 min.

### Medications

At enrolment, all participants declared salbutamol use on demand (2 × 100 μg.d^−1^ just before exercise or in case of asthma symptoms). None of them reported salbutamol dose higher than 400 μg.d^−1^ over the past year before enrolment in this study. Four declared a treatment to control asthma (see *Subjects* in the *Results* section). All subjects had to abstain from using any medicine, including SABA, 2 weeks before the pharmacokinetic session.

The protocol of the study is detailed in Fig. 1. At least one week before the rest pharmacokinetic session (D1, Fig. 1), all participants received a full salbutamol aerosol inhaler and a valve holding chamber. To follow salbutamol consumption, the weight of two puffs was arbitrarily defined by weighing each inhaler before and after 4 puffs and the weigh difference divided by two.

Each participant received written and oral instructions to inhale salbutamol three times a day (2 puffs/each time). Participants were asked to inhale 200 μg salbutamol three times daily scheduled for 3 consecutive days (D1-3, D1-2, D1-1). This scheme of salbutamol inhalation was reproduced on the 4th consecutive day designed as the rest pharmacokinetic session (D1). From D1-3 to D2 (exercise pharmacokinetic session, next day after D1), salbutamol was administered in the same conditions. Participants were asked to bring back the inhaler at D2. At the end of the exercise pharmacokinetic session, the inhaler was weighted again and the difference in weight was used to control salbutamol administration.

Despite subjects enrolled in the study were not under a daily salbutamol inhalation treatment scheme, regular and pre-exercise inhalation of salbutamol were proposed for the study to mimic a treatment scheme by SABA that might be proposed as a part of the global treatment (SABA and addition of a controller) for more severe asthmatics. The dose of 200 μg for each of the three daily inhalations was defined because this dose is close to the maximal dose recommended before exercise (400 μg) while this dose would be usual for mild asthmatic subjects.

### Urine sample

During the rest and exercise pharmacokinetic sessions, urine was collected at baseline (*T*_*0*_) and at 30 min, 2 h (i.e., just after the 90-min intense exercise in the case of the exercise pharmacokinetic session), 4 h and 6 h after the first dose of 200 μg of salbutamol. Participants were encouraged to drink water regularly to favour urine excretion for sampling. Night urine was collected at home by the participants in a dark container and delivered to our laboratory kept out from light. For each time point, the total urine sample volume was recorded and two 30 mL aliquots were stored at −80 °C until analysis.

### Bioanalytical analysis of urine samples

Urine samples were pre-treated according to the current procedures used by anti-doping laboratories to detect total salbutamol (i.e., salbutamol and its glucuronide) [15]. Salbutamol-D9 was used as internal standard. After extraction from urine by solid-phase extraction, salbutamol concentration was assessed by hydrophilic interaction chromatographic and liquid chromatography with atmospheric pressure chemical ionization mass spectrometry. Bioanalytical analyses were performed at the Pharmacokinetic and Toxicological Laboratory of Toulouse Hospital (France). This laboratory is accredited by COFRAC, the French accreditation body, under the European norm n°1589.

Urine salbutamol concentration values were corrected to the urine density, as previously recommended [9]. The maximum urine concentration of salbutamol for each participant and for each experimental session was defined as the highest value for each session (C_u_max-rest and C_u_max-ex, in ng.mL^−1^, were labeled for resting and exercising session respectively). For each participant, the highest of these values was defined as the C_u_max (the maximum urine concentration of salbutamol that could be detected for each participant, regardless the specific rest or post-exercise conditions).

### Statistical analysis

Sample size calculation to fit well-powered analysis in order to test our main hypothesis was based on the salbutamol concentration distribution in urine samples described by Tomlinson *et al*. [16] and with the aim of computing the confidence interval for salbutamol concentration in urine according to the general methodological recommendations by Gardner and Altman [17] and the recommendations edited by an Expert Panel on Theory of Reference Values from the International Federation of Clinical Chemistry and Laboratory Medicine [18]. We thus needed to include 12 subjects to assume a precision of 85 and a 95 % confidence level for the confidence interval. The confidence intervals of salbutamol distribution were computed for 95 and 99 % confidence levels.

For bivariate analysis, the normality of distribution of quantitative variables was tested using the skewness and kurtosis test (sktest). As some variables differed from the normal distribution, bivariate analysis was performed using the Wilcoxon matched-pairs ranks test or the Friedman’s test for repeated variables. Bivariate analysis was carried out with a significance level <0 · 05. For repeated variables, a Bonferroni adjustment of this significance level was performed to conclude on the significance of the corresponding multiple comparisons (corresponding to multiple time points).

All statistical analyses were performed with the Stata 6 · 0 software (Stata Corporation, College Station, TX).

## Results

### Subjects

Twelve subjects (mean age: 29 years ± 8 · 6 SD) were recruited. Baseline lung function assessed by whole-body plethysmography was in the normal range for all the ubjects (Table 2). Mean PD20 was 587 μg (±535 SD). Functional aerobic assessment (VO_2_max, MAP and maximum heart rate) and the mean power sustained during the exercise pharmacokinetic session are presented in Table 3. According to the ATS categorization of bronchial hyper-responsiveness (BHR), four participants (33 · 3 %) had moderate to severe BHR, two (16 · 7 %) had mild BHR and six (50 %) were borderline. Four subjects declared a treatment to control asthma: 10 mg.d^−1^ montelukast (*n* = 1), 50 μg.d^−1^ salmeterol + fluticasone (*n* = 1), 12 μg.d^−1^ formoterol (*n* = 1) or 12 μg.d^−1^ formoterol + budenoside (*n* = 1). As required, these subjects stopped their standard treatment 2 weeks before the first pharmacokinetic session (D1).

**Table 2:** Distribution of baseline respiratory characteristics of the subjects (*n* = 12 male participants)

	Minimum	Median	Maximum	Mean	SD
TLC	99	115	133	116	12 · 0
Forced vital capacity	97	118	130	117	11 · 6
FEV_1_	93	110	125	110	9 · 1
FEV_1_/FVC ratio	75	78	92	79	6 · 7
PD20; μg	50	535	1500	587	535

Abbreviations: *TLC* total lung capacity, *FVC* forced vital capacity, *FEV*_*1*_ one-second forced expired volume, *FEV*_*1*_ */FVC ratio* Tiffeneau-Pinelli index. Data for TLC, FVC, FEV_1_ and FEV_1_/FVC ratio are % of the corresponding predicted value

**Table 3:** Distribution of the results of functional aerobic assessment of the subjects (*n* = 12 male participants; VO_2_max: maximal oxygen uptake; MAP: maximal aerobic power; SD: standard deviation)

	Minimum	Median	Maximum	Mean	SD
VO_2_max; mL.min^−1^	2800	3800	4700	3858	340
VO_2_max; mL.min^−1^.kg^−1^	41	59	67	57	8 · 4
MAP; watts	210	305	340	294	40
MAP/weight; watt.kg^−1^	3.3	4.4	5.1	4.3	0 · 6
Maximum heart rate; beats.min^−1^	177	192	214	193	9 · 9
Mean power during exercise session; watts	160	225	250	215	31 · 6
Mean power during exercise session; ratio to MAP %	62 · 1	74	81 · 0	73 · 2	5 · 7

All the subjects practiced cycling at competitive level and were well-trained endurance athletes as indicated by their VO_2_max and MAP/kg values.

### Urine density

**Fig. 2:**
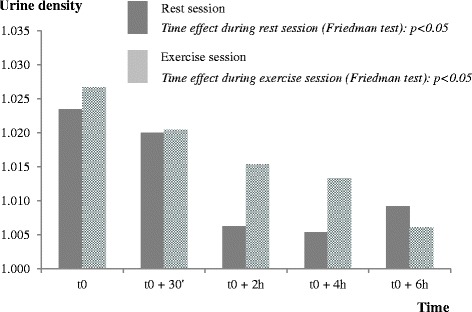
Urine density variation during the rest (dark grey columns) and exercise (light grey columns) pharmacokinetic sessions. Stratified on rest or exercise session, repeated measures were assessed using the Friedman’s test: *p* <0.05 for the time variation of urine density during rest and exercise sessions

As participants were encouraged to drink water ad libitum to ensure diuresis (required for urine sampling), urine density significantly decreased from the beginning to the end of each session (Fig. 2).

### Salbutamol urinary concentration

**Fig. 3:**
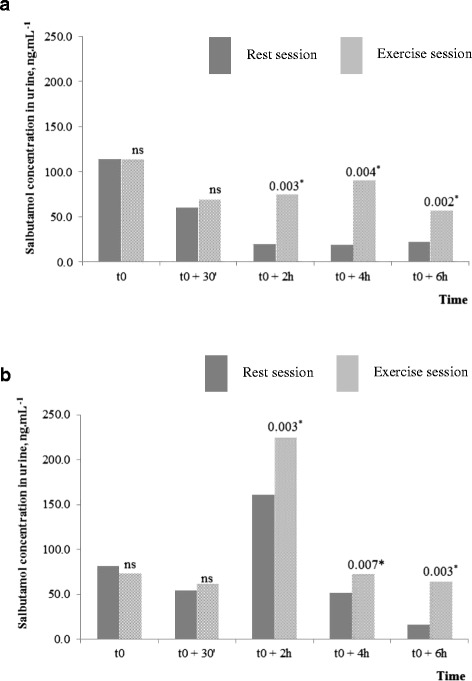
**a** Salbutamol urine concentration (ng.mL^−1^) before (dark grey columns) and after correction (light grey columns) for urine density during the rest pharmacokinetic session. Bivariate comparisons (Wilcoxon sign rank test) between corrected and uncorrected time-stratified values. Bonferroni correction of the significance level: * *p* < 0.01. **b** Salbutamol urine concentration (ng.mL^−1^) before (dark grey columns) and after correction (light grey columns) for urine density during the exercise pharmacokinetic session. Bivariate comparisons Wilcoxon (sign rank test) between corrected and uncorrected time-stratified values. Bonferroni correction of the significance level: *p < 0.01

Figure 3a and b present the distribution of salbutamol concentration in urine during the rest and exercise sessions, respectively. Table 4 presents the distribution of C_u_max-rest, C_u_max-ex and C_u_max and their confidence interval. C_u_max was observed in ten participants during the exercise session and in nine after the exercise bout. The maximal concentration was measured at 2:00 pm (i.e., 4 h after the exercise bout). Based on the upper value of the 95 and 99 % confidence intervals, the threshold value of urine salbutamol for anti-doping control could be stricter, at 327 · 7 and 372 · 4 ng.mL^−1^ respectively.

**Table 4:** Distribution of the urine density corrected maximum urine concentration of salbutamol at rest (C_u_max-rest), after the acute exercise bout (C_u_max-ex) and regardless these specific rest or post-exercise conditions (highest of these specific values for each participant, C_u_max)

	C_u_max-rest	C_u_max-ex	C_u_max
	*ng.mL* ^*−1*^	*ng.mL* ^*−1*^	*ng.mL* ^*–1*^
*Minimum*	46 · 9*	47 · 4	66 · 0
*Median*	134 · 7*	116 · 4	127 · 2
*Maximum*	276 · 7*	507 · 2	507 · 2
*Mean*	144 · 6	208 · 9	218 · 8
*Standard deviation*	74 · 7	179 · 4	171 · 3
*95 % CI*	94 · 4 – 194 · 7	94 · 9 – 322 · 8	110 · 0 – 327 · 7
*99 % CI*	73 · 2 – 215 · 6	48 · 1 – 369 · 7	65 · 2 – 372 · 4

95 % CI: 95 % Confidence Interval. *Wilcoxon test between rest and exercise values: *p* = 0.04

## Discussion

One could argue that there is no need to change the rules but rather to educate athletes, coaches and the health care providers who care about the proper use of SABAs rather than to prevent athletes from participating. However, sanction against cheaters remains a key point to fight against doping in sport and we need to improve the judgment criteria before adopting any disciplinary sanction against an athlete. By using conditions in accordance with the experts’ clinical and safety guidelines for asthma management in athletes undergoing an intense exercise bout, our study demonstrates that the urine salbutamol concentration threshold could be lowered to redefine the rule supporting the decision to sanction an athlete for salbutamol abuse. Under a minimal inhaled salbutamol dose regimen, our study demonstrates that a urine salbutamol concentration threshold of 507 ng.mL^−1^ could be used to redefine the rule supporting the decision to sanction an athlete for salbutamol abuse. This threshold is very far from the actual WADA threshold defined for a not recommended inhaled salbutamol dose. Even a “statistically” more stringent threshold could be proposed if anti-doping instances planned to consider the upper limit of the 99 % confidence interval of salbutamol concentration in urine, and not the absolute maximum value detected. Our proposal to implement a new threshold is supported by methodological points supporting scientific based bases for anti-doping policy. However, this proposal is also closely related to some other methodological points that make our proposal too conservative to be applied for the heterogeneous population of asthmatic athletes.

Recently, Elers et al. highlighted methodological concerns that should be taken into account in scientific studies to support anti-doping policies against salbutamol abuse. Salbutamol dosing regimen, athletic status and testing conditions (rest/exercise) were previously shown to influence the pharmacokinetics of inhaled salbutamol [19]. This methodological concern was also stressed by the Joint Task Force of European Respiratory Society and the European Academy of Allergy and Clinical Immunology: “Although treatment of EIB has been extensively studied in asthmatic subjects, it was not so in athletes with EIB and it is not known whether athletes with EIB respond similarly to subjects with classical allergic or nonallergic asthma” [20]. Such a methodological defect can also be highlighted in recent studies supporting the WADA restrictions to detect an abuse of inhaled formoterol according to formoterol concentration in urine [21]. Our study is the first to follow this new methodological approach as asthmatic and trained subjects were tested during a bout of intense exercise. Our study could have been done in controls subjects defined as non-asthmatic athletes to clearly identify the impact of the asthmatic status on salbutamol pharmacokinetic in urine but we were limited by organizational conditions. Elers tried to give an answer to this question after administration of a single high inhaled dose of salbutamol (800 μg) [22]. However he selected nonathletic subjects as controls and despite he found no influence of asthma on urine salbutamol concentration, this question remains unresolved to implement some scientific data that could be useful to enhance specificity of the detection of salbutamol abuse in asthmatic but also nonasthmatic athletes.

Moreover, urine density correction of urine salbutamol concentration must be considered to minimize the effect of this biological condition on the anti-doping judgment criteria [19]. While this correction was not applied to urine salbutamol concentration proposed in the article by Berges et al. [11] that was used to define the actual upper threshold in the WADA prohibition list, we considered this potent bias and we corrected the urine salbutamol concentration for urine density. This correction is of interest to take into account the state of hydration because dehydration alters the urine specific density, for example during intense and prolonged exercise, thus favoring an overestimation of urine salbutamol [9].

According to these two methodological points, our study mirror field sports practice and it could help to implement some required medical and scientific based bases for doping control management [23].

The urine concentration threshold derived from our study after a daily minimal dose of inhaled salbutamol to prevent EIB (3 × 200 μg) is twice lower than the actual threshold of 1000 ng.mL^−1^ proposed in the WADA prohibited list to sanction salbutamol abuse. However, this “legal” threshold is based on pharmacokinetic data after a daily dose of 1600 μg inhaled salbutamol, i.e. 8 fold the total dose sequentially administrated in our study. Despite regular daily chronic use of salbutamol as a controller is not recommended by current guidelines, we justify the three times daily inhalation of salbutamol scheduled for the present study to prevent asthma symptoms in the four patients who declared a daily treatment to control asthma. These patients were asked to abstain from using this treatment 2 weeks before the pharmacokinetic session to avoid a potent influence of such treatments on salbutamol kinetic, as previously suggested for inhaled corticosteroids for example [24]. The dosing regimen was justified to ensure homogeneity of the pharmacological condition proposed in all volunteers. After such a preliminary study, we highlight that influence of a treatment to control asthma on inhaled salbutamol kinetic in exercise condition and in asthmatic athletes would be of interest to optimize sensitivity and specificity of salbutamol abuse detection in sport field conditions.

At the present time, experts’ guidelines for EIB do not explicitly state an optimal or minimal dose of salbutamol but ATS EIB guidelines do cite a reference which states that a patient should be instructed to use two (200 μg) to four (400 μg) puffs of an inhaled SABA 30 to 60 min before exercise [7]. Thus, considering that more severe asthmatics undergoing very intense exercise for prolonged periods might require additional doses of salbutamol (i.e. 4 total puffs, 400 μg) before exercise, we are aware that our pharmacokinetic data on salbutamol urine concentration are conservative for implementation as a threshold for anti-doping legacy. After 400 ug inhaled salbutamol, the resulting salbutamol urine concentration could have been higher than the C_u_max reported in our study. However, we can argue that we selected the lowest dose recommended by experts in respect to the corresponding asthma severity of the patients from our study. Moreover, even if we had considered the highest dose proposed by clinical experts (400 μg), this dose would have been very far from the maximal inhaled dose of salbutamol authorized by the WADA list (1600 μg) and our clinical based approach questions about ethical and medical safety of the very high dose of inhaled salbutamol authorized by WADA. Indeed, we kept in mind that regular use of BAs can lead quickly to tolerance (tachyphylaxis), reduction of their bronchoprotective effect [2, 20] and that high metrics of salbutamol use are supposed to be good markers of current asthma control, risk of future severe exacerbations and increased risk of future extreme salbutamol overuse [25]. We could have evaluated the beneficial and adverse effects of the daily regular minimal dose of salbutamol inhaled but the design of our study did not evaluate these effects of salbutamol use as a basis for determining a threshold to detect inhaled salbutamol abuse. Thus, one being not able to exclude that adverse effects associated with high inhaled dose of salbutamol are related to either more severe asthma or an adverse response to SABA, we questioned about ethical and medical safety of a very high inhaled salbutamol dose (like 1600 μg.j^−1^) as a treatment scheme in athlete while the athlete is exposed to hyperventilation during intense and prolonged exercise, a potent condition for asthma occurrence. Sports practice should not be exempted from avoiding common factors associated with asthma exacerbation, such as allergens and pollutants for allergic asthma, but also hyperventilation of cold and dry air for EIB, especially when high dose of inhaled salbutamol could be “required”. This assertion could prevent athletes to inhale high doses of salbutamol, not “inadvertently” for doping, but “intentionally” in respect to the safety of medical and environmental considerations. However, in the case of asthma crisis, a high dose of salbutamol could be inhaled by athletes in a day, up to 1600 μg as authorized by WADA. If the crisis occurs out of competition, this high dose might be acceptable because it could be associated with the control of the environmental conditions, particularly avoidance of ventilatory conditions that triggers EIB. Under this medical consideration, we suggest to consider that anti-doping legacy to detect salbutamol abuse could be based on two salbutamol urine concentration, one that could apply out of competition (a high dose of inhaled salbutamol could be authorized) and one that could apply during a competition (a high dose of inhaled salbutamol wouldn’t be authorized).

A last point supporting the conservative aspect of our study is related to the methodological choice that limited the study to men. This choice was due to multiple pharmacological variations in women related to menstrual cycle. Like us, most of the previous studies to assess the pharmacokinetic of salbutamol were selectively conducted only in men. The study supporting the actual WADA urine salbutamol concentration threshold is one of these studies. Thus, future studies should target a female cohort of asthmatic athletes to implement the results to all asthmatic athletes.

## Conclusion

Based on medical considerations and methodological concerns, our study provides some evidence about the need to revise the actual WADA maximum inhaled salbutamol dose authorized and the urine salbutamol concentration threshold used to detect and sanction salbutamol abuse in athletes. Our revised urine salbutamol concentration threshold is based on medical evidence on the recommended daily inhaled dose of salbutamol and dosing regimens. In the case of an asthma attack or in the presence of recurrent symptoms, the dose of inhaled salbutamol could be increased but we argue that athletes should also be excluded from risky situations for EIB, like hyperventilation that is common during competition. Accordingly, we propose to consider that further studies should be planned to define the upper confident threshold of salbutamol concentration in urine in asthmatic athletes, in competition and out of competition, after inhalation of an upper limit-low dose (400 μg) or a high dose (1600 μg) salbutamol. Notwithstanding the pharmacokinetic dimension of the problem, WADA prohibited list should also base the recommendations on the maximum daily dose of inhaled salbutamol on best practice approaches for asthma management.

## Abbreviations

WADA: World Anti-Doping Agency
IOC: International Olympic Committee
EIB: Exercise induced bronchospasm
ATS: American Thoracic Society
BA: beta2-agonist
SABA: Short-acting beta2-agonist
GINA: Global Initiative for Asthma
COPD: Chronic obstructive pulmonary disease
APS: Aerosol Provocation System
FEV1: Forced expiratory volume in 1 second
PD20: Provocative dose 20 %, μg
VO_2_: Oxygen consumption debt, L.min^−1^
MAP: Maximal aerobic power, watt
BHR: Bronchial hyper-responsiveness
